# Discovery of physalin biosynthesis and structure modification of physalins in *Physalis alkekengi* L. var. *Franchetii*

**DOI:** 10.3389/fpls.2022.956083

**Published:** 2022-10-10

**Authors:** Liyuan Qu, Chunli Gan, Xiaoling Cheng, Congcong Lin, Yanli Wang, Libo Wang, Jian Huang, Jinhui Wang

**Affiliations:** Department of Pharmaceutical Chemistry, College of Pharmacy, Harbin Medical University, Harbin, China

**Keywords:** Physalins, plant metabolomics, biosynthetic pathways, Daodi medicinal materials, *Physalis alkekengi* L var. *franchetii* (mast.) Makino

## Abstract

Physalins, active ingredients from the *Physalis alkekengi* L. var. *franchetii* (*P. alkekengi*) plant, have shown anti-inflammatory, antioxidant and anticancer activities. Whereas the bioactivity of physalins have been confirmed, their biosynthetic pathways, and those of quite a few derivatives, remain unknown. In this paper, biosynthesis and structure modification-related genes of physalins were mined through transcriptomic and metabolomic profiling. Firstly, we rapidly and conveniently analyzed physalins by UPLC-Q-TOF-MS/MS utilizing mass accuracy, diagnostic fragment ions, and common neutral losses. In all, 58 different physalin metabolites were isolated from *P. alkekengi* calyxes and berries. In an analysis of the physalin biosynthesis pathway, we determined that withanolides and withaphysalins may represent a crucial intermediate between lanosterol and physalins. and those steps were decanted according to previous reports. Our results provide valuable information on the physalin metabolites and the candidate enzymes involved in the physalins biosynthesis pathways of *P. alkekengi*. In addition, we further analyzed differential metabolites collected from calyxes in the Jilin (Daodi of *P. alkekengi*) and others. Among them, 20 physalin metabolites may represent herb quality biomarkers for Daodi *P. alkekengi*, providing an essential role in directing the quality control index of *P. alkekengi*.

## Introduction

**Figure 1:**
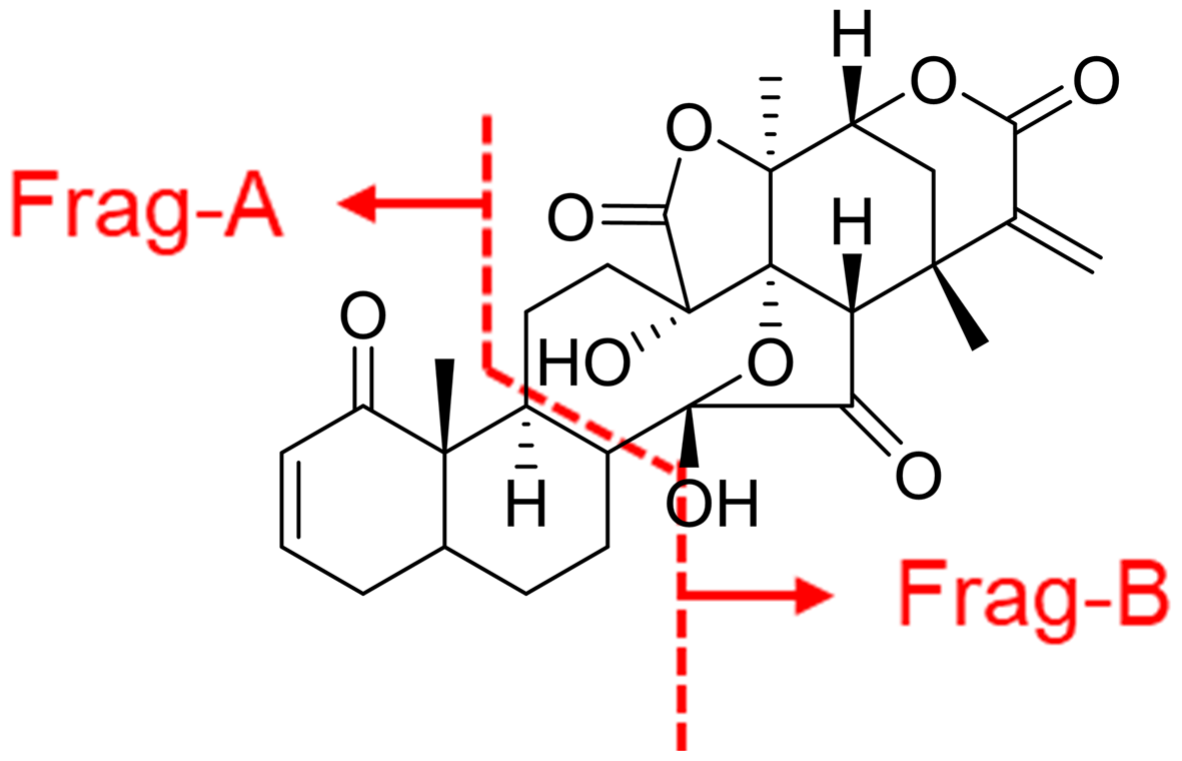
The skeleton of physalins. The skeleton of physalins was split into two parts, Frag-A and Frag-B. Frag-A is comprised of 2 six-membered ring molecules, where 1-C is the carbonyl group. Frag-B is comprised of four parts, where 27-C and 14-OH are likely to form rings or annular structures.

Medicinal plants provide humans with a valuable source of potential therapeutic agents. Historically, physalis plants such as *Physalis alkekengi* L. var. franchetii (*P. alkekengi*) have been used to treat inflammation for centuries(Li et al., 2018). Their anti-inflammatory properties are based on the production of physalins(Shu et al., 2016). The physalin skeleton has been found to be an extraordinarily complex, 13–14 secosteroid skeleton incorporating a nine-membered carbocycle derived from a steroidal ring fused to a six-membered ring with δ-lactone (Sun et al., 2017). These complexes are the structural basis of physalin, which has been studied for more than half a century. Differences between physalins, replace substituents with those bearing different electromagnetic charges. Other major differences are located in 27-C, such as in physalin A and physalin G. Based on this, the skeleton of physalins was split into two parts, Frag-A and Frag-B (Figure 1). Frag-A is comprised of 2 six-membered ring molecules, where 1-C is the carbonyl group. Frag-B is comprised of four parts, where 27-C and 14-OH are likely to form rings or annular structures. Physalins are mainly found in plants of the genus Physalis (Li et al., 2014; Wu et al., 2020; Xu et al., 2013), and they are biologically active, that is, they can inhibit tumor growth (Zhu et al., 2016), suppress lymphocyte proliferation (Ding et al., 2020), and inhibit inflammatory processes (Wu et al., 2012). Physalins are a potential source of targeted drug libraries due to their variety and complexity in structures and types of compounds.

Plants possess a diverse array of metabolic products arising from both primary and secondary metabolisms. Plant metabolomics involves the comprehensive analysis of plant metabolites and secondary metabolism (Last et al., 2007; Sawada et al., 2009; Urano et al., 2010). The plant metabolome is regarded as the bridge connecting the genome with the phenome (Luo, 2015). Based on this, the correlation between chemical composition and biosynthesis could be established by plant metabolomics. It offers a source of information about the physiological status of plants (Oikawa et al., 2015). The main strategies in plant metabolomics studies are metabolic fingerprinting and metabolite profiling (Dunn and Ellis, 2005). Spectral characteristics, sensitivity, and quantification of metabolomics are primarily addressed by mass spectrometry (MS) and nuclear magnetic resonance (NMR) spectroscopy. Recent years have seen a rapid advancement in liquid chromatography and mass spectrometry systems that have driven plant metabolomics (Sawada and Yokota Hirai, 2013).

**Figure 2:**
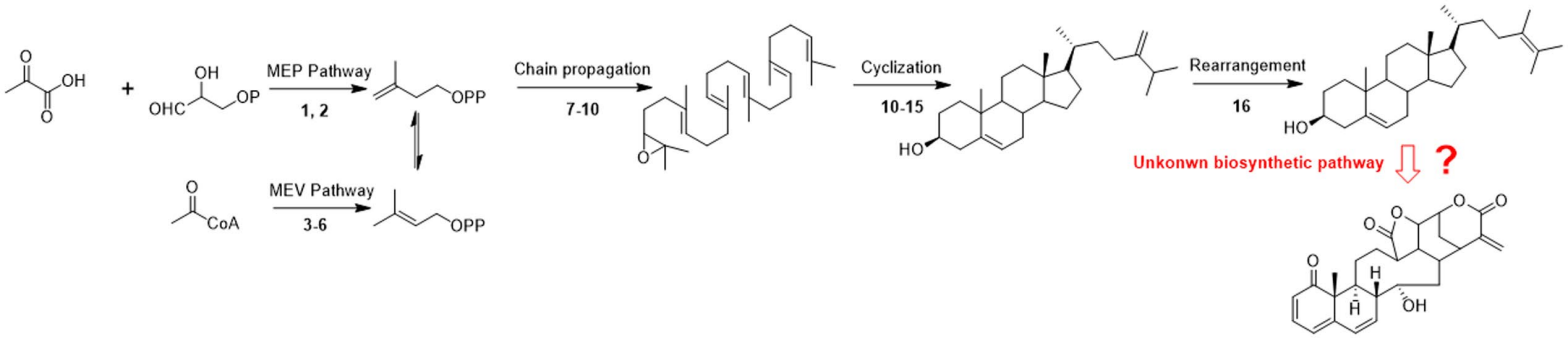
Possible pathways of physalin biosynthesis in previous studies. 1-deoxy-d-xylulose-5-phosphate synthase; (2) 1-deoxy-d-xylulose-5-phosphate reductase; (3) 3-hydroxy-3-methylglutaryl-coenzyme A reductase; (4) mevalonate kinase; (5) phosphomevalonate kinase; (6) mevalonate diphosphosphate decarboxylase; (7) farnesyl diphosphate synthase; (9) squalene synthase; (10) squalene epoxidase; (11) cycloartenol synthase; (12) sterol methyl transferase 1; (13) sterol-4α-methyl oxidase 1; (14) cycloeucalenol cycloisomerase; (15) obtusifoliol-14-demethylase; sterol-4α-methyl oxidase 2; (16) stero-Δ-24-isomerase.

Previous studies of physalin biosynthetic pathways have concentrated on biosynthetic-related genes. The first reported study on biosynthesis of physalins was carried out by Fukushima et al. (2016), where 24-methyldesmosterol was designated as a key intermediate in physalin biosynthesis (Fukushima et al., 2016). Relevant studies were continually advanced, withanolide were designated as the new key intermediate physalin biosynthesis (Knoch et al., 2018; Yang et al., 2022b). However, the key steps for progress from 24-methyldesmosterol to physalins is still unexplored (Figure 2).

The purpose of this study is to follow-up research based on the previous studies. Further data mining of their RNA Sequencing data was performed to interpret the physalin biosynthesis pathway in *P. alkekengi* (Fukushima et al., 2016). Our view of physalin biosynthesis was based upon the structural characterization of physalin in *P. alkekengi*. Plant metabolomics helps to dissect primary and secondary metabolites. Based on the cleavage rules of physalins, our research team used UPLC-Q-TOF-MS/MS to detect structural diversity in physalins. In the present study, 58 physalin metabolites were identified. Through data mining using the online database, a further analysis of the biosynthesis pathway for physalins is presented here. Collectively, the modification of physalin skeletons is achieved by desaturation, methylation, hydroxylation, epoxidation, cyclization, chain elongation, and glycosylation, etc. Finally, we compare the differences of major physalins between geographically diverse calyxes of *P. alkekengi*.

## Materials and methods

### Chemicals and reagents

Acetonitrile (MS grade) and formic acid (LC–MS grade) were all purchased from Thermo Fisher Scientific Corporation. Other chemicals and reagents were analytical grade and commercially available. The standard substances physalin A, physalin D, physalin G, physalin L and physalin O (purity greater than 97%) were isolated and purified by our laboratory, and the method used for preparation of these standard substances has been described previously (Lin and Wang, 2011). All standards were dissolved in methanol and then diluted to a final concentration of 10 μg/ml. Before use, all standard solutions were stored in glass storage vial at –20°C.

### Plant material and sample preparation

*Physalis alkekengi* fruit collection took place across China in May and June of 2020. The fruits were rapidly isolated, immediately after being collected, and all samples were put on ice until they were transported to –80°C for storage. In total, 18 different regional fruits were collected. Sample information is shown in Table. 1.

**Table 1:** Geographical distribution of sample information.

Samples No	Sample	Sample type	Origin (province)	Sampling time
20,210,506-PAC-HJL-01	*Physalis alkekengi* L.var.	Whole fruit	Heilongjiang	October
20,210,506-PAC-JL-01	*Physalis alkekengi* L.var.	Whole fruit	Jilin	October
20,210,506-PAC-NL-01	*Physalis alkekengi* L.var.	Whole fruit	Liaoning	October
20,210,506-PAC-SD-01	*Physalis alkekengi* L.var.	Whole fruit	Shandong	August
20,210,506-PAC-ZJ-01	*Physalis alkekengi* L.var.	Whole fruit	Zhejiang	August
20,210,506-PAC-JL-02	*Physalis alkekengi* L.var.	Whole fruit	Jilin	August
20,210,506-PAC-JL-03	*Physalis alkekengi* L.var.	Whole fruit	Jilin	August
20,210,506-PAC-HN-01	*Physalis alkekengi* L.var.	Whole fruit	Hunan	August
20,210,506-PAC-JL-04	*Physalis alkekengi* L.var.	Whole fruit	Jilin	August
20,210,506-PAC-JL-05	*Physalis alkekengi* L.var.	Whole fruit	Jilin	August
20,210,506-PAC-JL-06	*Physalis alkekengi* L.var.	Whole fruit	Jilin	August
20,210,506-PAC-JL-07	*Physalis alkekengi* L.var.	Whole fruit	Jilin	August
20,210,506-PAC-JL-08	*Physalis alkekengi* L.var.	Whole fruit	Jilin	August
20,210,506-PAC-JL-09	*Physalis alkekengi* L.var.	Whole fruit	Jilin	August
20,210,506-PAC-JL-10	*Physalis alkekengi* L.var.	Whole fruit	Jilin	August
20,210,506-PAC-JL-11	*Physalis alkekengi* L.var.	Whole fruit	Jilin	August
20,210,506-PAC-HN-02	*Physalis alkekengi* L.var.	Whole fruit	Hunan	August
20,210,506-PAC-HLJ-02	*Physalis alkekengi* L.var.	Whole fruit	Heilongjiang	August

The calyxes and berries were separated manually, and extracted by ultrasonication. Each sample (~ 1 g) was extracted with 10 ml of methanol at room temperature (25°C) for 30 min, the liquid supernatants were further processed by solid-phase extraction (SPE) by the following steps: The SPE was activated with methanol and then with water. After loading 1 ml of sample, the SPE was washed with 1 ml of methanol, and the filtrate was combined and retained for subsequent compositional analysis.

### LC–MS/MS conditions

The metabolic profile of P. alkekengi calyxes and berries were determined by UPLC-Q-TOF MS/MS (Waters Corporation, Milford, MA, United States). Chromatography was performed on a UPLC system (Waters Corp., United States) with an autosampler at 4°C. The separations were carried out on an ACQUITY UPLC^®^ BEH C18 column (1.7 μm, 2.1 × 50 mm, Waters Corporation, United States). The column temperature was maintained at 45°C. The analysis was performed with gradient elution using (A) methanol and (B) water with 0.1% formic acid as the mobile phase: 0–4.5 min, 10–50% A; 4.5–11.5 min, 50–98% A; a work-loop with no injection was carried out before assaying samples for equilibration of the column. The flow rate was set to 0.4 ml/min. Throughout the analysis, all samples were kept at 4°C. A Waters AcquityTM Mass Spectrometer (Waters Corp., Milford, CT, United States) was connected to the UPLC system *via* an electrospray ionization (ESI) interface. The capillary voltage and sample cone voltage, source offset voltage and source temperature of positive modes were 3.0 kV, 40 V, 80 V, and 100°C, respectively. The desolvation temperature was 250°C. The cone and desolvation gas flow rates were 50 l/h and 600 l/h, respectively. The data acquisition rate was set at 0.2 s/scan, with a 0.1 s (0.02 interscan time) interscan delay. MS data were collected in centroid mode from 50 to 1,000 Da. MS data were obtained by using MSE acquisition mode. For accurate mass acquisition, a lock-mass of leucine enkephalin was used to ensure accuracy through the whole MS analysis. Data were obtained by Masslynx 4.1 (Waters Corp., United States).

### Data mining analysis

The data mining analysis was carried out using the commercial software Progenesis QI (Version 2.3, herein after referred to as QI) and UNIFI v1.9.3 software (Waters Corp.).

#### Identification of metabolites

Structural identification of the secondary phytochemicals (physalins) was based on different cleavage rules of physalins. We used several strategies, such as diagnostic fragmentation screening and fragment monitoring, during the process.

#### Statistical analysis

The raw data were imported into QI to be processed. This included peak annotation and normalization. Analysis of all metabolites between groups was performed. Multivariate statistical analysis was performed using orthogonal partial least squares-discriminant analysis (OPLS-DA) and variable importance in projection (VIP) of each metabolite was determined (EZinfo 3.0, an internal software program of Progenesis QI).

## Results and discussion

### Data mining strategies for rapid discovery and characterization of physalins

**Figure 3:**
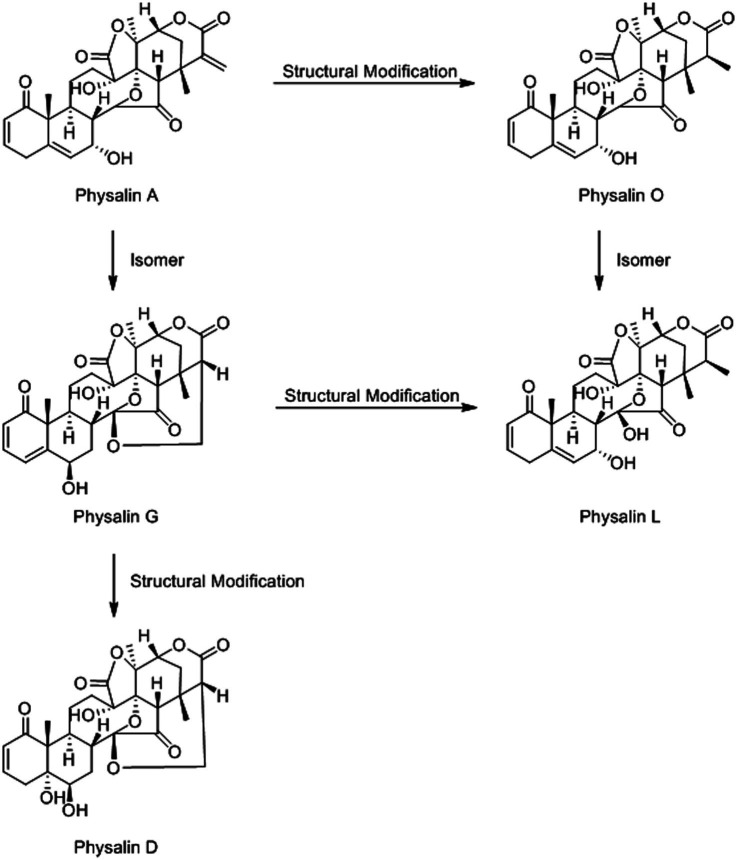
The structural association among physalin A and other physalins. Those five physalins could be categorized into two types: the differences among physalin A, physalin D and physalin G is mainly at the ring between 27-C and 14-OH. Besides, there two morphological of 27-C among physalin A, physalin O and physalin L. And, the detailed differences are in the quantity and distribution of the different functional groups.

The structure of physalin is complex and covers a wide variety of structural deformation(Sun et al., 2017). This makes mass spectrometric identification difficult. To solve this problem, we employed a high-throughput method for physalin identification. Five physalin standards (physalin A, D, G, L and O) were investigated to ascertain their MS/MS fragmentation behaviors, providing a basis for structural elucidation of physalins. The structural association among physalin A and others is shown in Figure 3.

**Figure 4:**
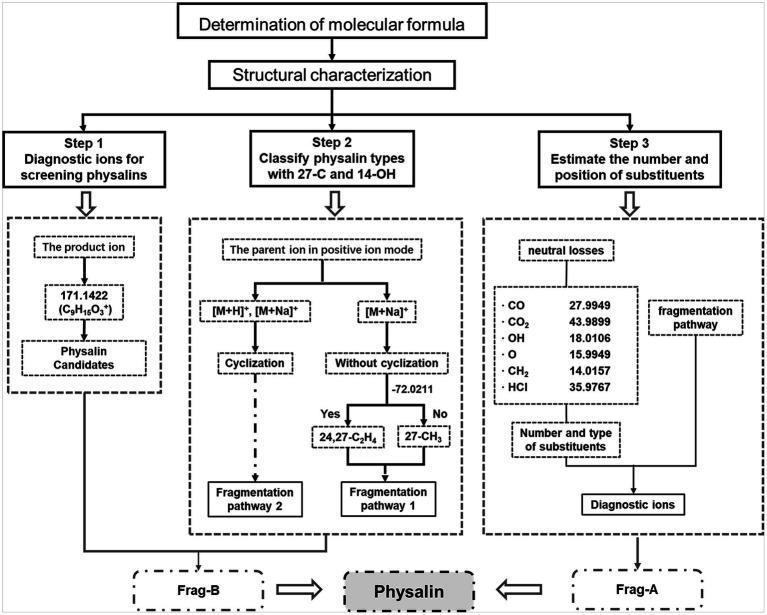
Data mining strategies for rapid discovery and characterization of physalins. Three key steps were developed for efficient characterization of non-targeted physalins using these strategies. (1) Initial screening of physalins using diagnostic ions. (2) Structure determination with 27-C and 14-OH. (3) Get feature information of the substituents.

Based on the MS behaviors of the physalin standards (described in detail in the Supplementary Material), a high-efficiency approach using LC–MS/MS together with a data mining strategy for rapid discovery and identification of non-targeted physalins in complex samples was proposed (Figure 4). Three key steps were developed for efficient characterization of non-targeted physalins using these strategies. The first key step was to screen physalins using diagnostic ions from Frag-B. 171.1422 was used as a diagnostic ion to screen precursor ions of physalin candidates. The second key step was to classify physalin types with 27-C and 14-OH. The cyclization between 27-C and 14-OH was judged by using parent ion data in positive ion mode. Then, the signature neutral loss of C_2_H_4_ and CO_2_ (−72.0211) from Frag-B was used for identify the types of 27-C. The third key step was to estimate the number and position of substituents using neutral losses and diagnostic ions from Frag-A. Generally speaking, the number and varieties of substituents were determined by the neutral fragment losses. Then, the positions of substituents were determined by comprehensive information in diagnostic ions which included substituents and signature fragments.

### The differential physalin metabolites in calyxes and berries of *Physalis Alkekengi*

**Figure 5:**
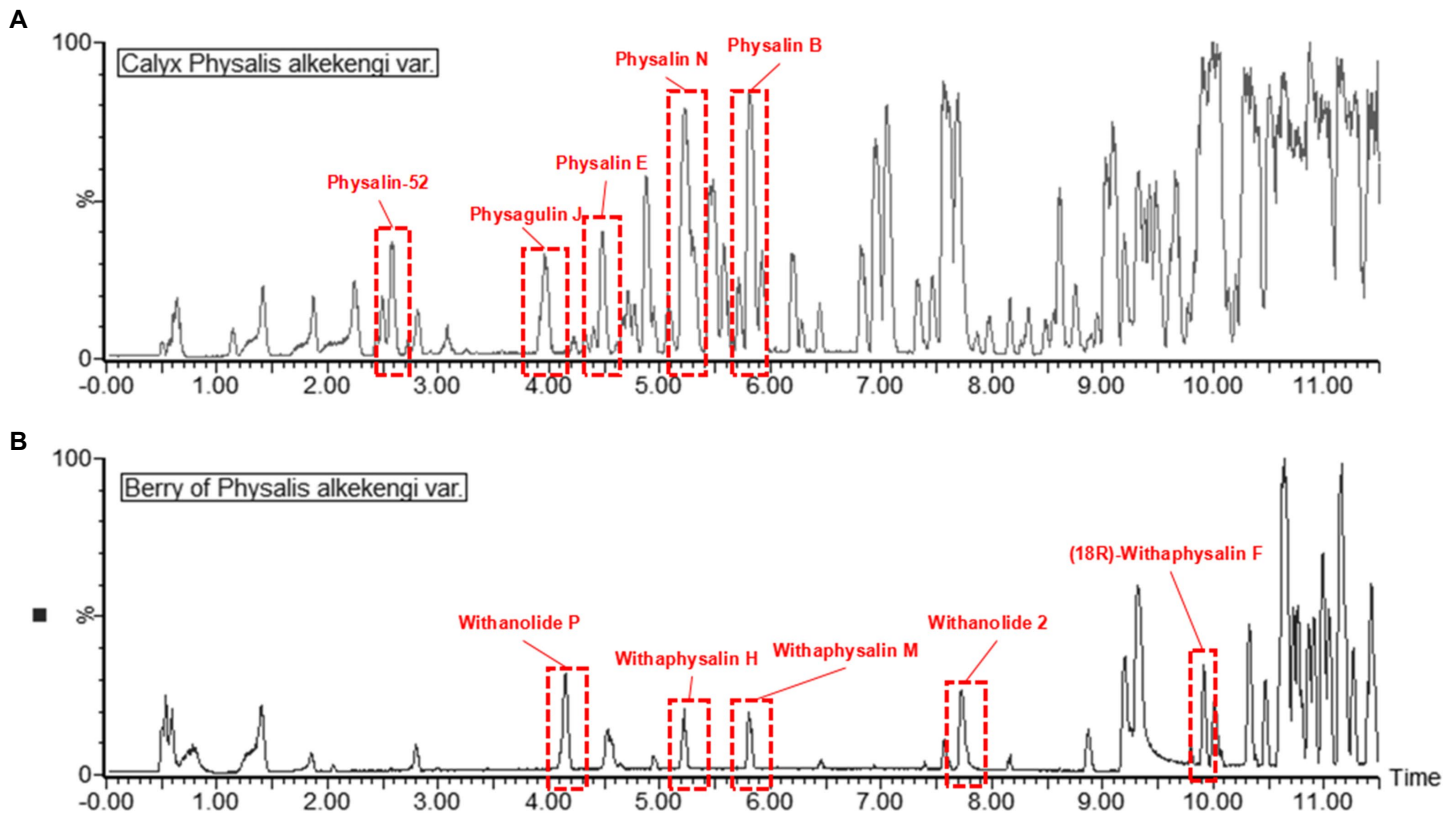
The BPI chromatograms of berry and calyx of *Physalis alkekengi* in positive ion mode. **(A)** The BPI chromatograms of calyx of *P. alkekengi*. **(B)** The BPI chromatograms of berry of *P. alkekengi.*

Primary metabolites were present in nearly every tissue and involved in synthesis of secondary metabolisms(Oikawa et al., 2015). Conversely, specialized secondary metabolism is generally limited to specific stages of organism development and occurs in specialized tissues for specific functions (Yazaki, 2005). It has been found that fruits are the main sources of physalins in *P. alkekengi* (Yang et al., 2022a). For this reason, fresh berries were isolated from whole fruit and were detected under the same conditions as calyxes (Figure 5). In all, 58 different physalin correlative metabolites were detected based on the identification strategy above in *P. alkekengi* (Table 2). In our data analysis, physalin correlative metabolites are divided into three main categories, which are withanolides, withaphysalins and physalins.

**Table 2:** Summary of the physalins metabolites identification results.

RT (min)	m/z	Mass error (ppm)	Formula	Compounds name	Pubchem ID
0.56	997.5366	2.50	C_29_H_38_O_7_	Physaminimin A	102,223,242
2.57	591.3134	7.61	C_32_H_46_O_10_	Physalin-52	60,005,230
3.70	565.2755	−0.38	C_31_H_42_O_8_	Physalin-09	140,492,210
3.93	531.2913	5.98	C_30_H_42_O_8_	Physagulin J	42,610,742
4.10	477.2638	−1.38	C_28_H_38_O_5_	Withanolide P	21,679,034
4.16	495.2717	0.23	C_28_H_40_O_6_	Vamonolide	13,939,886
4.18	577.2063	−1.38	C_30_H_34_O_10_	Physalin V	102,254,402
4.24	565.1662	−1.96	C_28_H_30_O_11_	Physalin-109	162,827,760
4.24	545.2044	4.76	C_28_H_32_O_11_	Physalin D	431,071
4.75	527.1919	−3.91	C_28_H_30_O_10_	Isophysalin A	57,387,832
4.75	567.1834	1.31	C_28_H_32_O_11_	Physalin E	13,962,952
4.90	583.1758	−4.51	C_28_H_32_O_12_	physalin-02	140,498,412
4.95	549.1731	−0.05	C_28_H_30_O_10_	Physalin J	44,426,449
4.99	513.1763	5.95	C_27_H_28_O_10_	Physalin-83	42,617,138
5.10	569.1986	0.88	C_28_H_34_O_11_	Physalin 2	140,646,467
5.10	529.2090	0.32	C_28_H_32_O_10_	Physalin L	146,156,484
5.17	553.2037	−2.92	C_28_H_34_O_10_	Physalin-17	91,351,492
5.17	513.2073	−9.06	C_28_H_32_O_9_	25,26-Epidihydrophysalin C	42,610,672
5.22	965.5406	−3.25	C_29_H_38_O_6_	Withaphysalin H	21,606,682
5.23	549.1733	5.48	C_28_H_30_O_10_	Physalin N	131,752,539
5.32	503.3031	3.33	C_29_H_42_O_7_	2,3-Dihydro-3beta-methoxy withaferin A	10,767,792
5.35	583.1800	5.81	C_28_H_32_O_12_	Physalin-66	58,594,640
5.59	549.1733	3.15	C_28_H_30_O_10_	Physalin F	49,864,133
5.75	505.2184	6.65	C_28_H_34_O_7_	Withaphysalin M	10,096,775
5.83	549.1728	0.28	C_28_H_30_O_10_	Physalin X	102,319,017
5.86	533.1787	1.44	C_28_H_30_O_9_	Physalin B	11,613,161
5.92	581.1993	−0.06	C_29_H_34_O_11_	Physalin I	131,751,562
5.93	549.1731	1.94	C_28_H_30_O_10_	Physalin Z	44,577,484
6.01	563.1926	7.01	C_29_H_32_O_10_	Physalin-120	163,183,929
6.05	535.1940	−6.00	C_28_H_32_O_9_	Physalin M	76,900,148
6.32	523.2286	0.39	C_28_H_36_O_8_	Withaperuvin E	85,089,867
6.71	489.2247	−3.10	C_28_H_34_O_6_	Withaphysalin A	14,216,298
6.96	533.1779	0.45	C_28_H_30_O_9_	Isophysalin B	22,748,169
7.12	549.1732	−5.08	C_28_H_30_O_10_	Physalin U	44,577,487
7.14	507.2344	0.35	C_28_H_36_O_7_	Withaphysalin N	11,752,064
7.14	467.2407	−0.52	C_28_H_34_O_6_	Withaphysalin A	14,216,298
7.31	489.2254	4.26	C_28_H_34_O_6_	Withaphysalin P	102,471,581
7.34	507.2354	1.50	C_28_H_36_O_7_	(18S)-Withaphysalin F	21,606,679
7.45	489.2250	−0.26	C_28_H_34_O_6_	Withaphysalin J	21,606,684
7.61	587.2869	6.92	C_32_H_42_O_10_	Withanolide 2	101,005,225
7.61	547.2141	−9.34	C_28_H_34_O_11_	Physalin-35	90,264,481
7.99	577.2307	−2.22	C_29_H_36_O_12_	Physalin G	11,017,274
9.11	487.2679	4.09	C_28_H_38_O_7_	Withanolide E	301,751
9.86	969.5062	−2.89	C_28_H_36_O_7_	(18R)-Withaphysalin F	21,606,678
10.20	625.3374	4.45	C_34_H_50_O_9_	Physapruin B	131,752,899
10.25	707.3253	4.74	C_36_H_50_O_14_	Physagulin E	10,009,993
10.45	523.2926	−3.32	C_28_H_42_O_9_	Physangulide	14,605,176
10.60	945.5771	−0.10	C_28_H_40_O_6_	Ixocarpanolide	14,605,184
10.64	523.2696	−7.43	C_29_H_40_O_7_	Withaphysalin R	102,471,583
10.80	961.5007	0.13	C_29_H_36_O_6_	Withaphysalin K	21,606,685
10.81	977.5583	−0.06	C_28_H_40_O_7_	14alpha-Hydroxyixocarpanolide	14,605,187
11.00	543.1897	1.92	C_28_H_30_O_11_	25-Hydroxy physalin F	73,350,641
11.15	531.2993	−4.97	C_30_H_42_O_8_	Physaminimin F	102,223,246
11.16	543.1882	4.82	C_28_H_30_O_11_	25-Hydroxy physalin J	73,346,012
11.20	977.5640	5.11	C_28_H_40_O_7_	3-HDH-withanolide F	135,887
11.41	909.5485	−2.69	C_28_H_38_O_5_	Withanolide P	21,679,034
11.44	933.4787	−0.12	C_28_H_34_O_6_	Withaphysalin D	101,293,641
11.44	569.3625	3.93	C_36_H_50_O_4_	Physanol A	102,067,218

**Figure 6:**
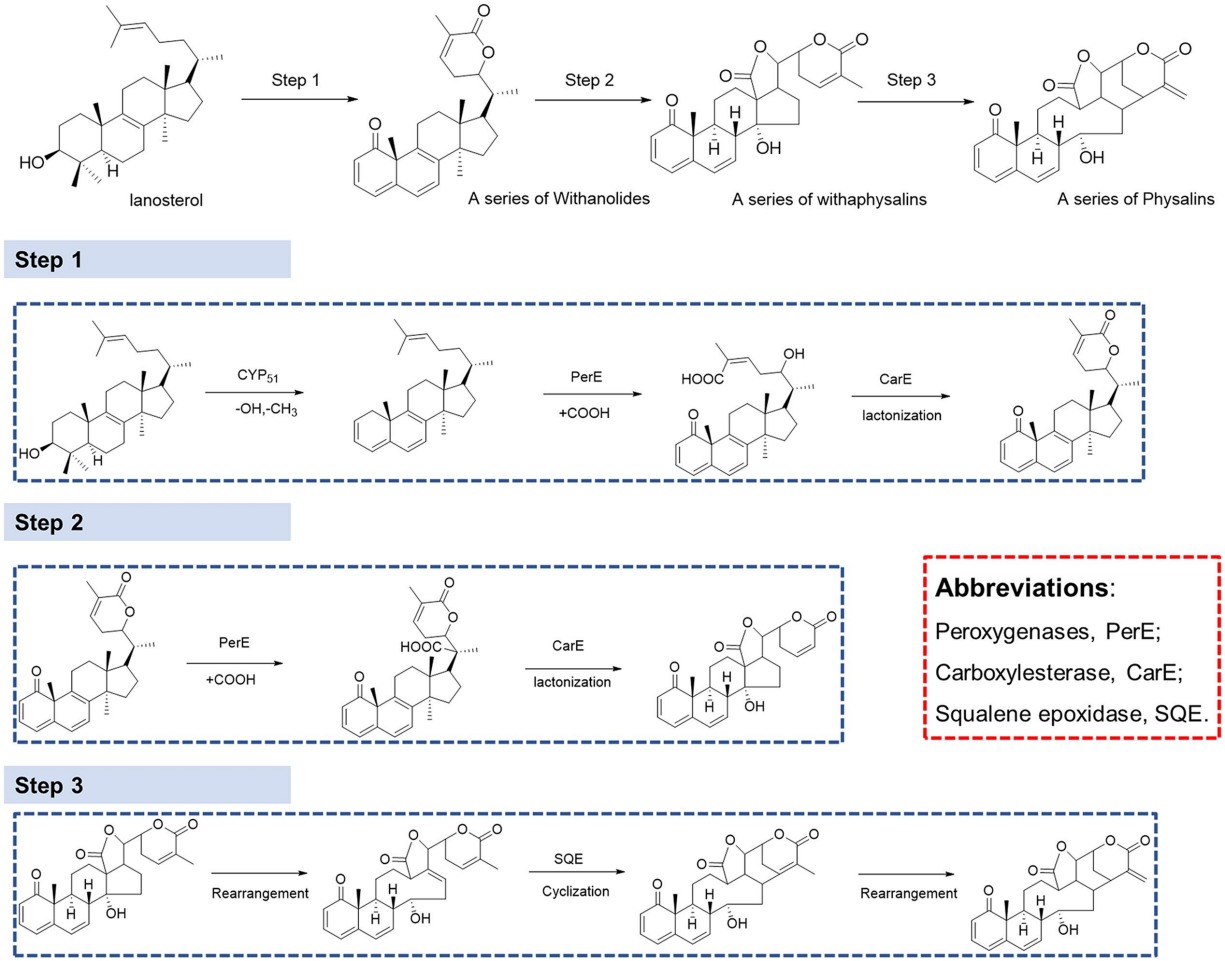
Heatmaps of physalin metabolites in berry and calyx. Physalins are more abundant in calyxes, and withanolides and withaphysalins are more abundant in berries, confirming the biosynthesis and structural modifications in fruit development.

In order to exclude interference from moisture while calculating the content for each ingredient, the quantitative analysis of all components was performed by area normalization using physalin D as the internal standard. Heatmaps of metabolites were drawn with the OmicStudio tools at https://www.omicstudio.cn. using unit UV and HCA (Figure 6). Using the identification criterion of the absolute Log2 FC ≥ 1, 46 physalin metabolite contents, including 32 upregulated metabolites and 14 downregulated metabolites, were found to be significantly different among the 58 physalin metabolites in berries vs. calyxes of *P. alkekengi*. We found that physalins are more abundant in calyxes, and withanolides and withaphysalins are more abundant in berries, confirming the biosynthesis and structural modifications in fruit development. The berries are responsible for physalin synthesis, whereas the calyx is responsible for physalin accumulation in *P. alkekengi* berries (Table 3).

**Table 3:** Candidate gene families associated with physalin biosynthesis.

Description	Enzyme codes	GOs
Demethylase	EC:1.14.11	C:cytosol; F:oxidoreductase activity, acting on paired donors, with incorporation or reduction of molecular oxygen, 2-oxoglutarate as one donor, and incorporation of one atom each of oxygen into both donors; P:oxidation–reduction process
Cytochrome p450	EC:1.14.13	F:oxidoreductase activity, acting on paired donors, with incorporation or reduction of molecular oxygen, NAD(P)H as one donor, and incorporation of one atom of oxygen; F:heme binding; F:iron ion binding; P:oxidation–reduction process
	EC:1.3.3.9	F:heme binding; F:oxidoreductase activity, acting on paired donors, with incorporation or reduction of molecular oxygen; F:monooxygenase activity; F:iron ion binding; P:oxidation–reduction process; F:secologanin synthase activity; P:indole biosynthetic process
	EC:1.14.13.77	F:heme binding; F:iron ion binding; P:oxidation–reduction process; F:taxane 13-alpha-hydroxylase activity; P:diterpenoid biosynthetic process
	EC:1.14.13.68	F:heme binding; F:iron ion binding; F:hydroxyphenylacetonitrile 2-monooxygenase activity; P:oxidation–reduction process; F:4-hydroxyphenylacetaldehyde oxime monooxygenase activity; P:tyrosine metabolic process
	EC:1.14.13.88	P:floral organ development; P:leaf formation; P:oxidation–reduction process; F:flavonoid 3′,5′-hydroxylase activity; P:regulation of meristem growth; P:positive regulation of organ growth; P:regulation of growth rate; F:heme binding; P:positive regulation of cell proliferation; F:iron ion binding; C:endoplasmic reticulum
	EC:1.14.14.1; EC:1.14.15.3	F:iron ion binding; F:heme binding; P:fatty acid metabolic process; F:alkane 1-monooxygenase activity; F:aromatase activity; P:oxidation–reduction process
	EC:1.14.15.3	P:fatty acid metabolic process; F:heme binding; F:iron ion binding; P:oxidation–reduction process; F:alkane 1-monooxygenase activity
	EC:1.14.13.76	F:heme binding; F:taxane 10-beta-hydroxylase activity; F:iron ion binding; P:oxidation–reduction process; P:diterpenoid biosynthetic process
	EC:1.14.13.21	F:identical protein binding; P:flavonoid biosynthetic process; F:flavonoid 3′-monooxygenase activity; C:plasma membrane; F:iron ion binding; F:heme binding; F:p-coumarate 3-hydroxylase activity; P:lignin biosynthetic process; P:coumarin biosynthetic process; C:endoplasmic reticulum; C:mitochondrion; P:oxidation–reduction process
	EC:1.14.13.36	F:identical protein binding; P:flavonoid biosynthetic process; F:5-O-(4-coumaroyl)-D-quinate 3′-monooxygenase activity; C:plasma membrane; F:iron ion binding; F:heme binding; F:p-coumarate 3-hydroxylase activity; P:lignin biosynthetic process; P:coumarin biosynthetic process; C:endoplasmic reticulum; C:mitochondrion; P:oxidation–reduction process
	EC:1.14.14.1	F:iron ion binding; F:heme binding; C:chloroplast membrane; C:chloroplast stroma; F:aromatase activity; P:oxidation–reduction process
	EC:1.14.14.1; EC:1.14.99	C:chloroplast envelope; F:zeinoxanthin epsilon hydroxylase activity; F:iron ion binding; F:heme binding; F:aromatase activity; P:oxidation–reduction process
	EC:1.6.2.4	C:endoplasmic reticulum membrane; P:response to oxidative stress; F:NADPH-hemoprotein reductase activity; F:iron ion binding; F:FMN binding; C:cytosol; P:response to abscisic acid stimulus; P:oxidation–reduction process; P:electron transport
	EC:1.8.1.7; EC:1.6.2.4	P:response to oxidative stress; C:cytosol; P:oxidation–reduction process; F:NADPH-hemoprotein reductase activity; C:peroxisome; P:cell redox homeostasis; P:glutathione metabolic process; F:glutathione-disulfide reductase activity; F:flavin adenine dinucleotide binding; F:NADP binding; C:endoplasmic reticulum membrane; F:iron ion binding; P:response to abscisic acid stimulus; F:FMN binding; P:electron transport; P:gluconeogenesis
Carboxylesterase	EC:3.1.1.3	F:triglyceride lipase activity; P:lipid catabolic process; P:glycerolipid metabolic process

### Biosynthetic pathway analysis of physalin metabolites in *Physalis Alkekengi*

**Figure 7:**
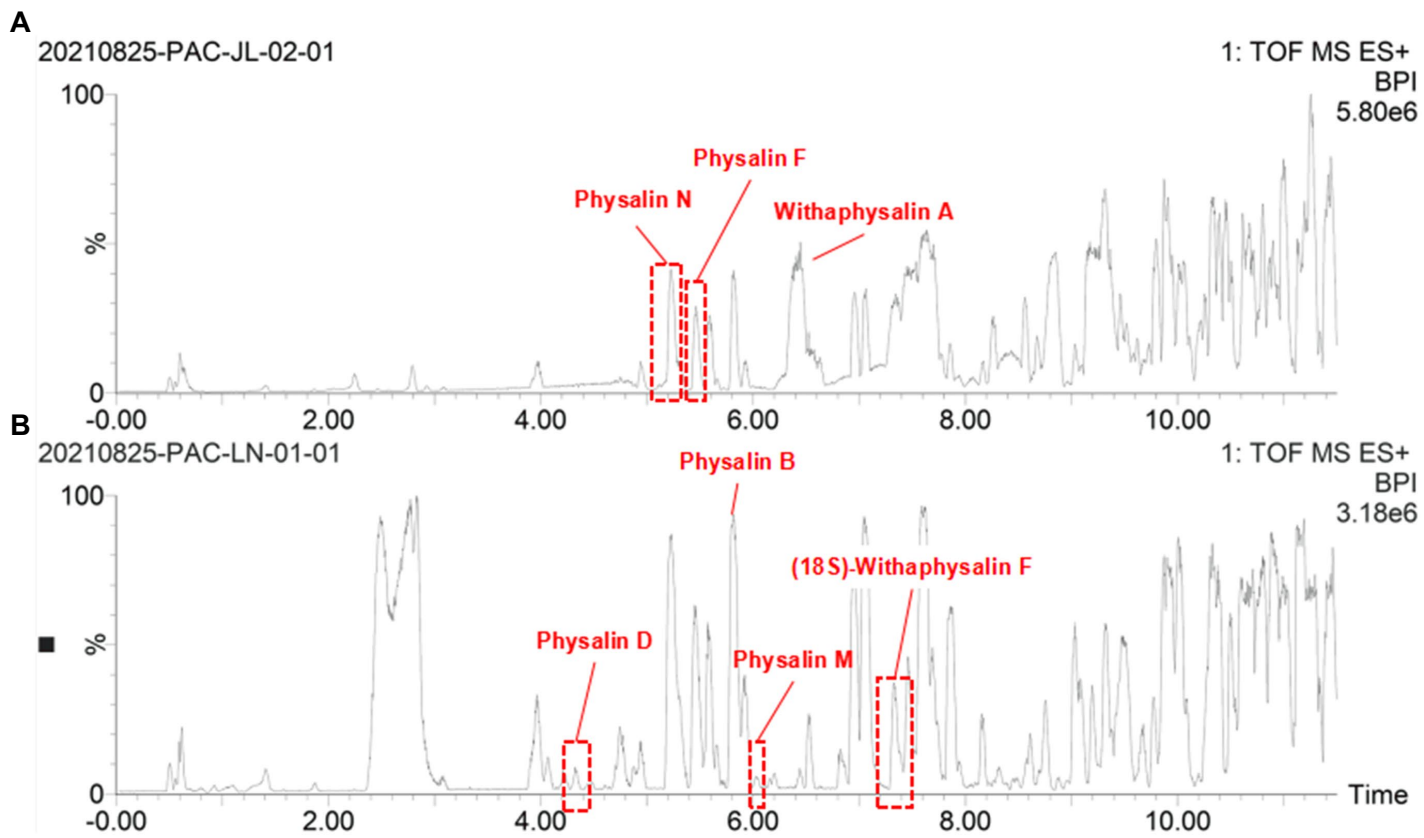
The BPI chromatograms of calyx form Jilin and Liaoning provience. **(A)** The BPI chromatograms of calyx of *P. alkekengi.* Form Jilin. **(B)** The BPI chromatograms of calyx of *P. alkekengi.* Form Liaoning.

It has been proven that physalin is well associated with mevalonate (MEV) and methylerythritol 4-phosphate (MEP) pathways, and that lanosterol is a key intermediate of the biosynthesis of physalins. However, there is still much that is unknown about the biosynthesis pathway from lanosterol to physalins(Fukushima et al., 2016). Withanolides and withaphysalins may represent an intermediate between lanosterol and physalins. To understand the molecular basis of physalin biosynthesis in *P. alkekengi*, transcriptomes were analyzed. We downloaded the Supplementary Material of transcriptomes from earlier literature (Fukushima et al., 2016), and molecular reclassification and identification of biosynthetic gene clusters for physalin metabolites. They discovered 40,090 UniGenes specific to *P. alkekengi*. Based on the results of physalin correlative metabolites, the synthesis of physalins can be divided into three steps (Figure 7).

In step 1, lanosterol generates withanolides with hydroxylation, lactonization, demethylation, and dehydrogenation, this is the first step toward the synthesis of physalins in plants. As steroidal lactones, withanolides are natural compounds that are formed when the oxo- and carbo-xylic groups of C22 and C26 are combined to form a six-membered lactone ring (Misico et al., 2011). In previous studies, it had been observed that methyltransferase and cytochrome P450 are main enzymes involved in the withanolides biosynthesis pathway (Gupta et al., 2015). Demethylation of lanosterol can be performed by demethylase hydroxylation or the hydroxyl radical damage of the methyl group (Mishra et al., 2019). Of all the genes, there are a total of 16 demethylases. Sterol 14α-demethylase (CYP51) belongs to the superfamily of cytochrome P450, which are the enzymes essential for sterol biosynthesis (Kim et al., 2005). CYP51 may potentially be the demethylase enzyme in this step. After demethylation, macrolactonization followed by ring-closing metathesis are the key steps. In this respect, peroxygenases and carboxylesterase represent a promising solution. This observation was further confirmed by observation that carboxylic acid derivatives of plant sterols were presented in our result. Peroxygenases are reported to catalyze hydroxylation (Babot et al., 2015). And carboxylase became the main enzyme responsible for the carboxylation reaction (Pontrelli et al., 2018; Roesler et al., 2021). Next, withanolides generate withaphysalins with hydroxylation and lactonization, which can be performed by peroxygenase and carboxylesterase enzymes. This is the second step toward the synthesis of physalins in plants. Finally, Michael rearrangement and cyclization reactions prompt the final synthesis of physalins, and is the third and paramount step toward the synthesis of physalins in plants. After physalin skeletons are formed, biochemical reactions (e.g., desaturation, methylation, hydroxylation, epoxidation, cyclization, chain elongation, and glycosylation) lead to various biochemical products of physalin skeletons.

### Physalin metabolites in geographically diverse calyxes of *Physalis Alkekengi*

**Figure 8:**
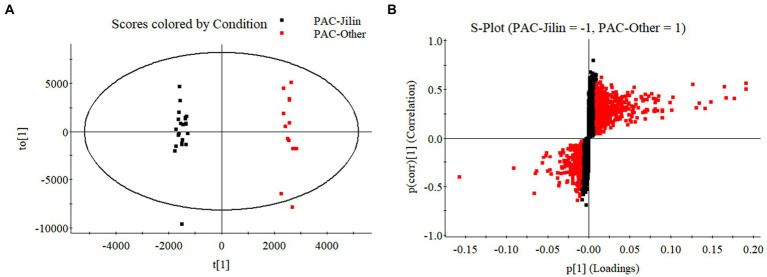
The OPLS-DA models distinguished geographically diverse calyxes of *P. alkekengi*. **(A)** The OPLS-DA models distinguished *P. alkekengi*. calyxes from Jilin and other proviences. **(B)** The S-Plot of potential distinguishing compounds.

Some traditional Chinese medicinal materials are produced in specific geographic regions with designated natural conditions and ecological environments. They are widely recognized as having beneficial clinical therapeutic effects, and are called Daodi medicinal materials, or geoherbs (Liu et al., 2020). Only the Jilin is regarded as an authentic Daodi production area for *P. alkekengi*. However, it is also found in others of China. In order to investigate the relationship between origins and differential physalin metabolites, we explored differences in the geographic locations of sample collection sites in different areas of China. Only 10 samples were collected from Jilin among all samples. Two batches of samples were divided into groups and their order was randomly assigned within batches. Then, primary phytochemicals and physalin metabolites were detected by UPLC-Q-TOF/MS/MS. MS/MS data was acquired in profile mode, and the MS profile of calyxes from Jilin and other had visible differences (Figure 8).

**Figure 9:**
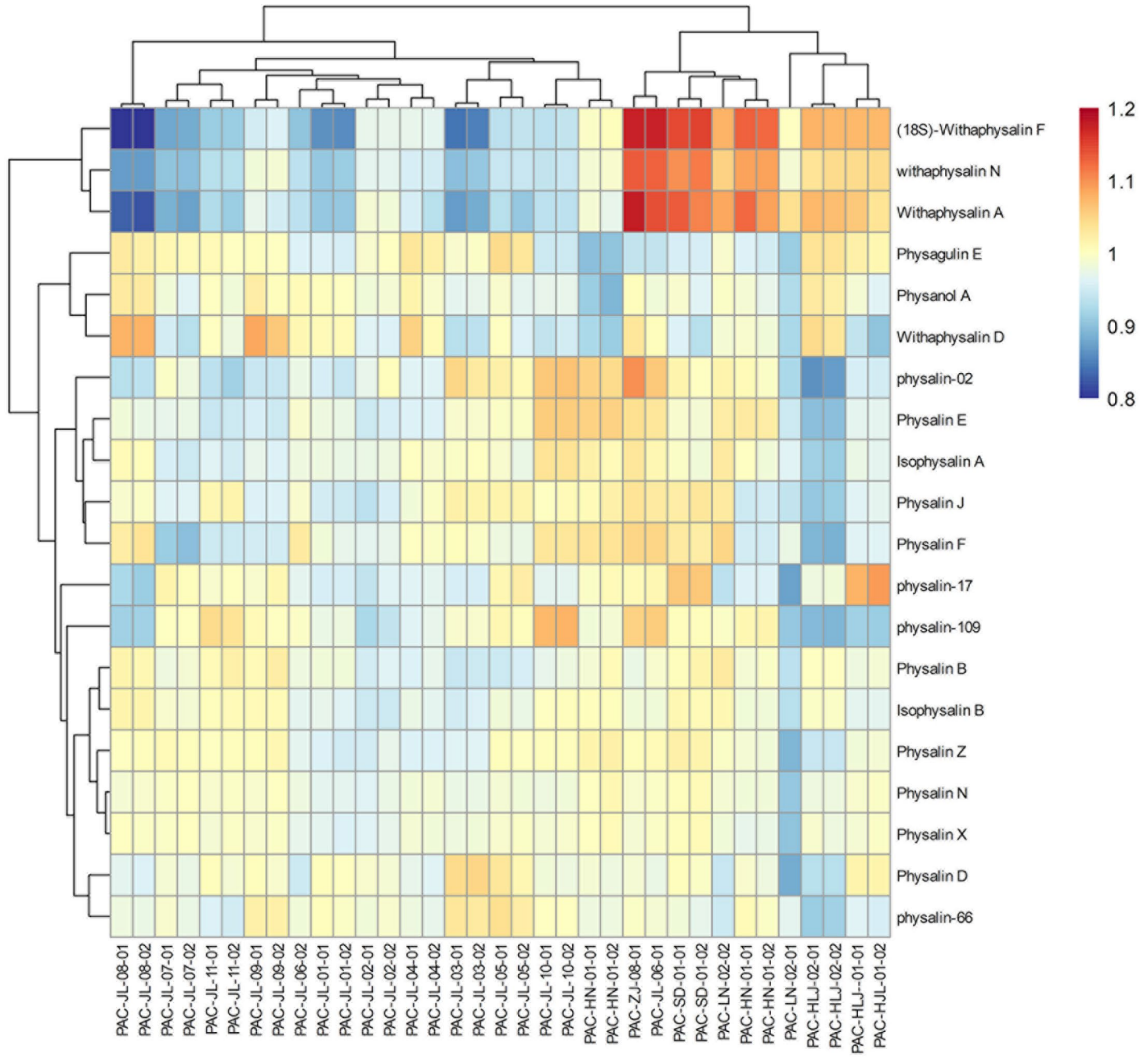
Heatmaps of physalin metabolites in geographically diverse calyxes of *P. alkekengi*.

Additionally, OPLS-DA models were created to separate those two groups further (Figure 9). The VIP was obtained by using OPLS-DA modeling. 20 physalin metabolites among all differential physalins show significant changes (evaluation criteria: VIP > 1), including 6 upregulated metabolites and 12 downregulated metabolites. Based on the accumulation pattern among various metabolites, heatmaps of metabolites were drawn with the OmicStudio tools at https://www.omicstudio.cn. using unit variance scaling (UV) and hierarchical cluster analysis (HCA). Calyx samples were also divided into two main groups within HCA. Specifically, samples obtained from Jilin had a significant difference compared to others. The samples from others were further divided into two subgroups. This suggests that those differential physalin metabolites can be employed to distinguish Daodi medicinal properties of *P. alkekengi* and others. In our study, the difference was mainly in expression of withaphysalins and physalins. Quantitative analysis of all components was performed by area normalization using physalin D as the internal standard. Among them, most physalins demonstrated significantly higher contents in the calyxes from Jinli vs. Others (Figure 10). However, withaphysalin N, withaphysalin A and (18S)-withaphysalin F demonstrated significantly lower contents (> 1.5-fold). Withaphysalins are important intermediates in physalin biosynthetic synthesis, and calyxes of *P. alkekengi* from others consumed more intermediates than those from Jilin. This may lead to fewer physalins being produced.

## Conclusion

We used metabolomics to identify the metabolic pathway involved in physalin biosynthesis. In all, 58 different physalin metabolites have been isolated from *P. alkekengi* calyxes and berries. In an analysis of the physalin biosynthesis pathway, we determined that withanolides and withaphysalins may represent crucial intermediates between lanosterol and physalins, and those steps were decanted according to previous reports. Our results provide valuable information on the physalin metabolites and candidate enzymes involved in the physalin biosynthesis pathways in *P. alkekengi*. In addition, we further analyzed differential metabolites between calyxes from Jilin (Daodi of *P. alkekengi*) and others. Among them, 20 physalin metabolites may represent herb quality biomarkers for Daodi P. alkekengi, providing an essential role in directing the quality control index of *P. alkekengi*.

## Data availability statement

The datasets for this study can be found in MetaboLights (https://www.ebi.ac.uk/metabolights/MTBLS5060).

## Author contributions

LYQ: design the experiment, completed the experiments and draft the manuscript. CLG: assisted editing and finalizing the manuscript. XLC: assist in completing the experiment. CCL: assisted in the completion of data processing. LBW: assisted in the completion of data processing. YLW: assisted in the sample collection and validation. JH and JHW: designed the experiments and supervised their completion. All authors contributed to the article and approved the submitted version.

## Funding

This work was supported by National Science and Technology Major Project of the Ministry of Science and Technology of China (no. 2018ZX09735005).

## Conflict of interest

The authors declare that the research was conducted in the absence of any commercial or financial relationships that could be construed as a potential conflict of interest.

## Publisher’s note

All claims expressed in this article are solely those of the authors and do not necessarily represent those of their affiliated organizations, or those of the publisher, the editors and the reviewers. Any product that may be evaluated in this article, or claim that may be made by its manufacturer, is not guaranteed or endorsed by the publisher.

## Supplementary material

SUPPLEMENTARY FIGURE 1 MS spectra of physalin A and fragmentation pattern of the protonated molecular ion. **(A)** molecular ion peak of physalin A. **(B)** fragmentation pattern of protonated molecular ion.

SUPPLEMENTARY FIGURE 2 MS spectra of physalin D and fragmentation pattern of the protonated molecular ion. **(A)** molecular ion peak of physalin D. **(B)** fragmentation pattern of protonated molecular ion.

SUPPLEMENTARY FIGURE 3 MS spectra of physalin G and fragmentation pattern of the protonated molecular ion. **(A)** molecular ion peak of physalin G. **(B)** fragmentation pattern of protonated molecular ion.

SUPPLEMENTARY FIGURE 4 MS spectra of physalin L and fragmentation pattern of the protonated molecular ion. **(A)** molecular ion peak of physalin L. **(B)** fragmentation pattern of protonated molecular ion.

SUPPLEMENTARY FIGURE 5 MS spectra of physalin O and fragmentation pattern of the protonated molecular ion. **(A)** molecular ion peak of physalin O. **(B)** fragmentation pattern of protonated molecular ion.

SUPPLEMENTARY FIGURE 6 Two fragmentation pathway in parent structure of physalins.
