# Supplementary figures and images for: Discovery of physalin biosynthesis and structure modification of physalins in Physalis alkekengi L. var. Franchetii
Source: Front Plant Sci. 2022 Oct 10;13:956083. doi: 10.3389/fpls.2022.956083 (PMC9589361; doi:10.3389/fpls.2022.956083)

**A**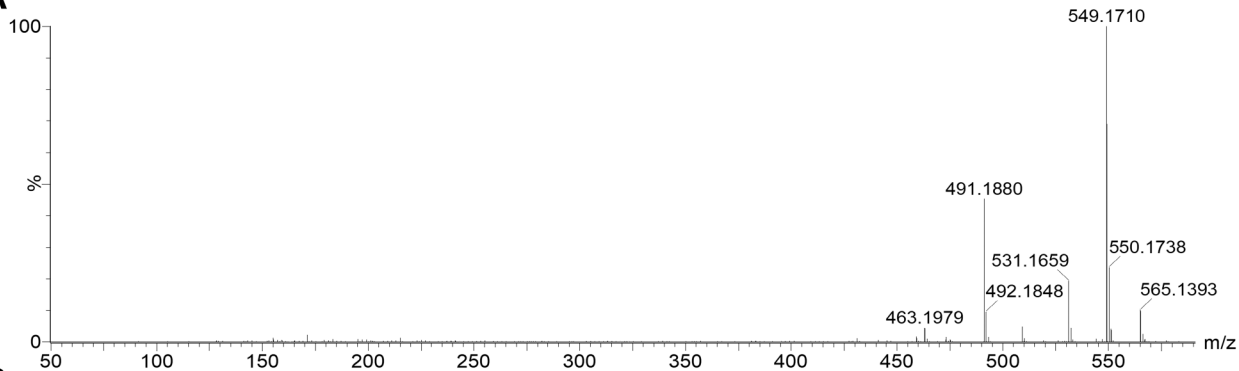**B**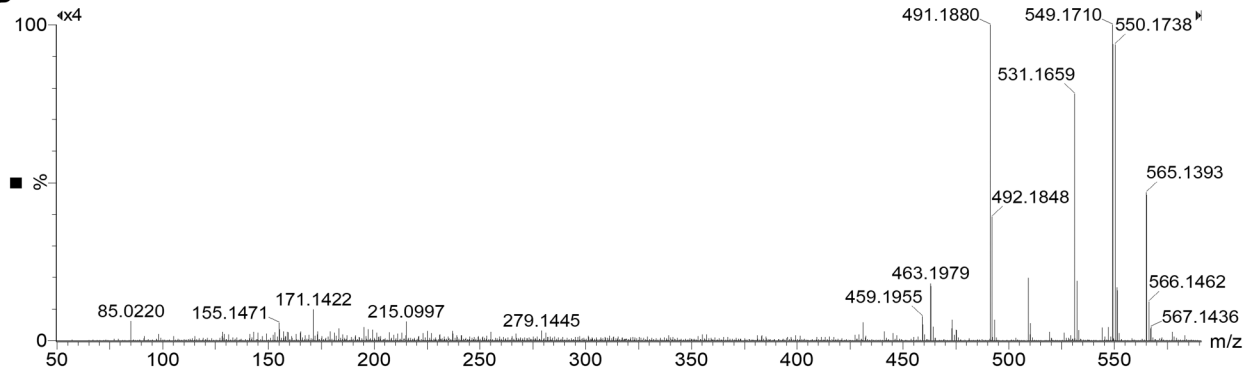

Supplement: SUPPLEMENTARY FIGURE 1 — MS spectra of physalin A and fragmentation pattern of the protonated molecular ion. (A) molecular ion peak of physalin A. (B) fragmentation pattern of protonated molecular ion. [file Image_1.PDF]

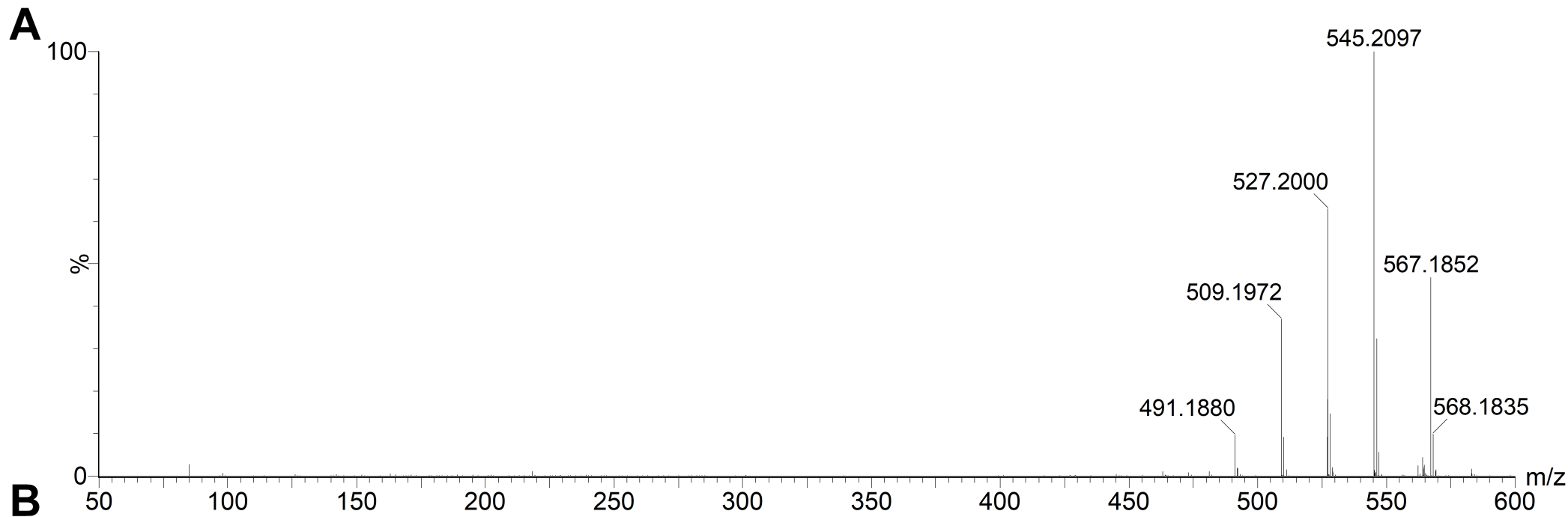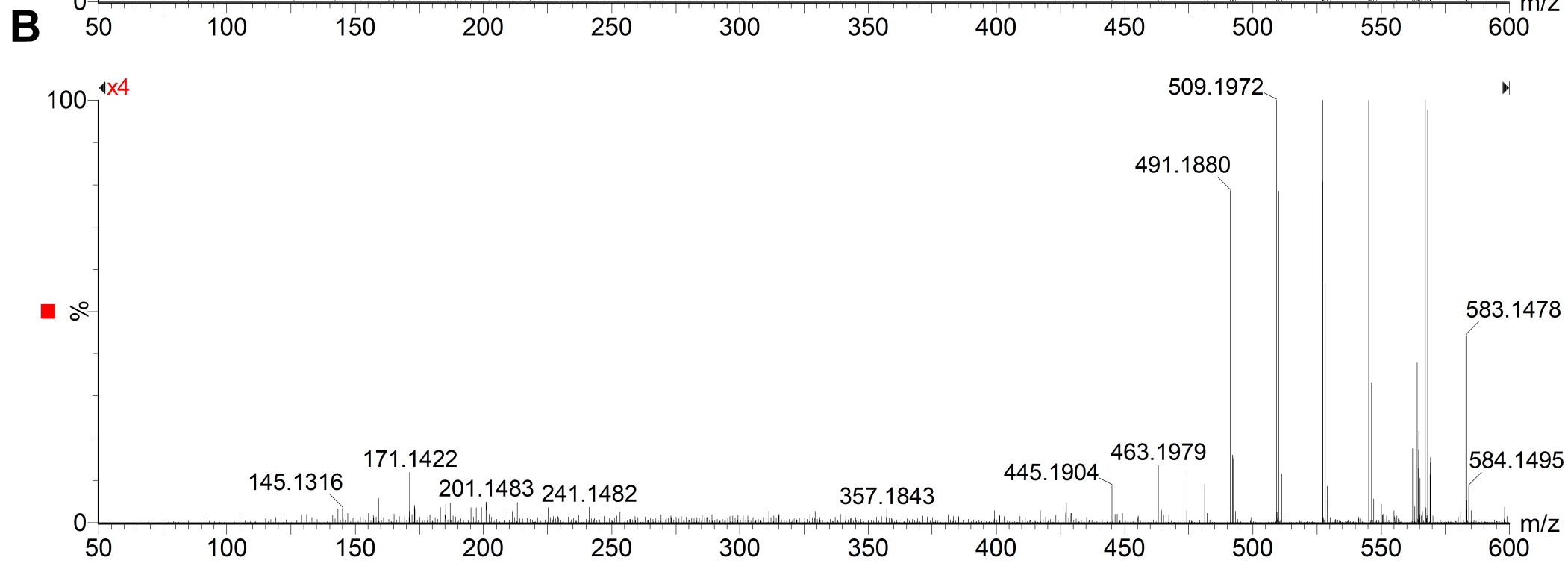

Supplement: SUPPLEMENTARY FIGURE 2 — MS spectra of physalin D and fragmentation pattern of the protonated molecular ion. (A) molecular ion peak of physalin D. (B) fragmentation pattern of protonated molecular ion. [file Image_2.PDF]

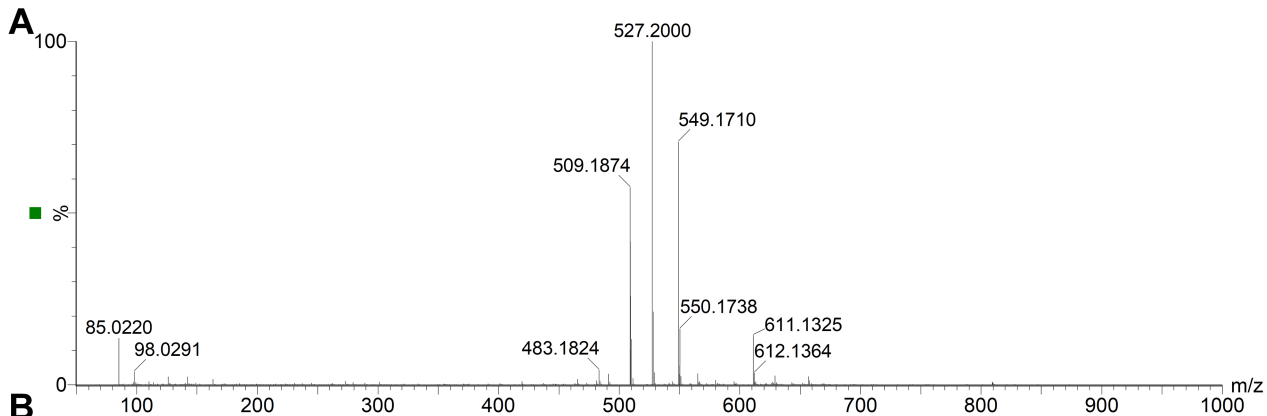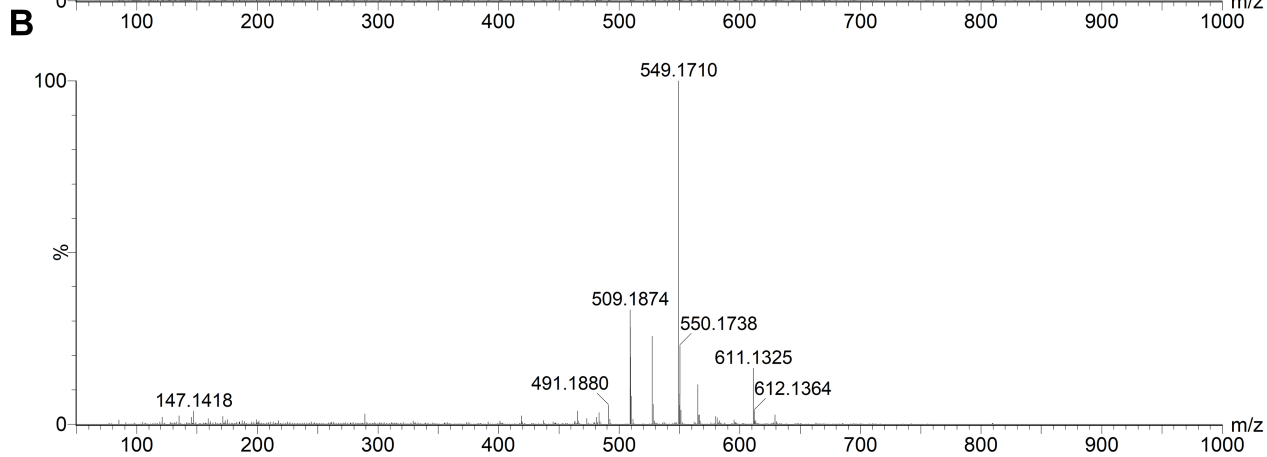

Supplement: SUPPLEMENTARY FIGURE 3 — MS spectra of physalin G and fragmentation pattern of the protonated molecular ion. (A) molecular ion peak of physalin G. (B) fragmentation pattern of protonated molecular ion. [file Image_3.PDF]

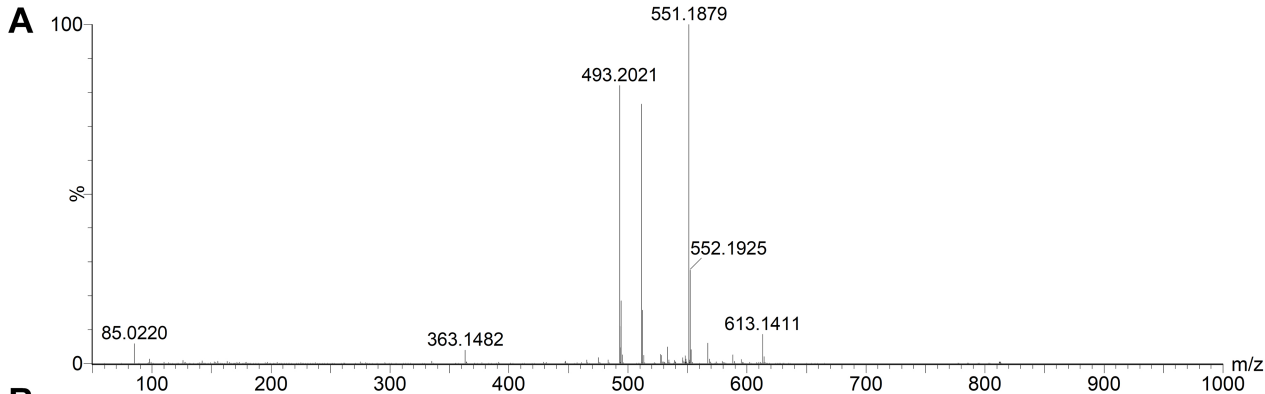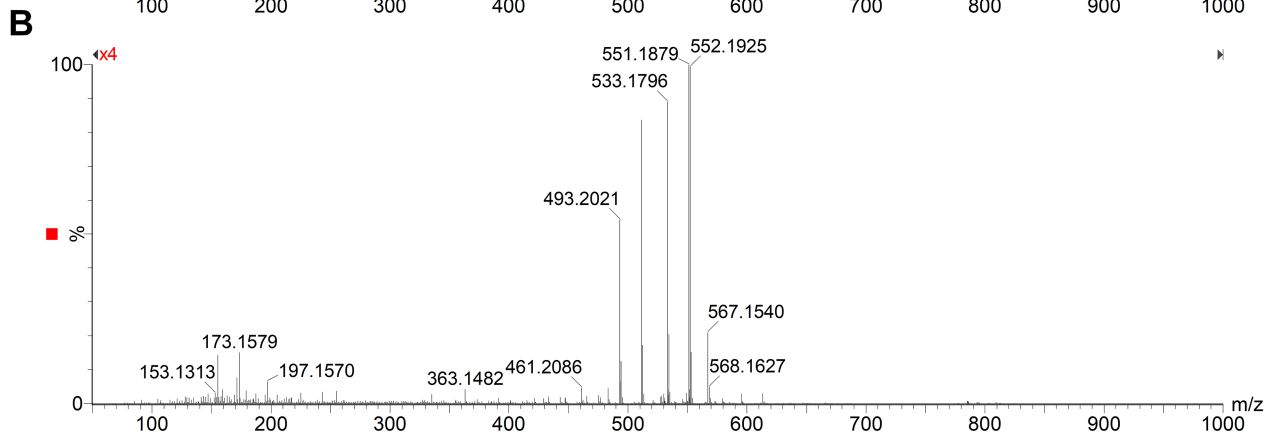

Supplement: SUPPLEMENTARY FIGURE 4 — MS spectra of physalin L and fragmentation pattern of the protonated molecular ion. (A) molecular ion peak of physalin L. (B) fragmentation pattern of protonated molecular ion. [file Image_4.PDF]

**A**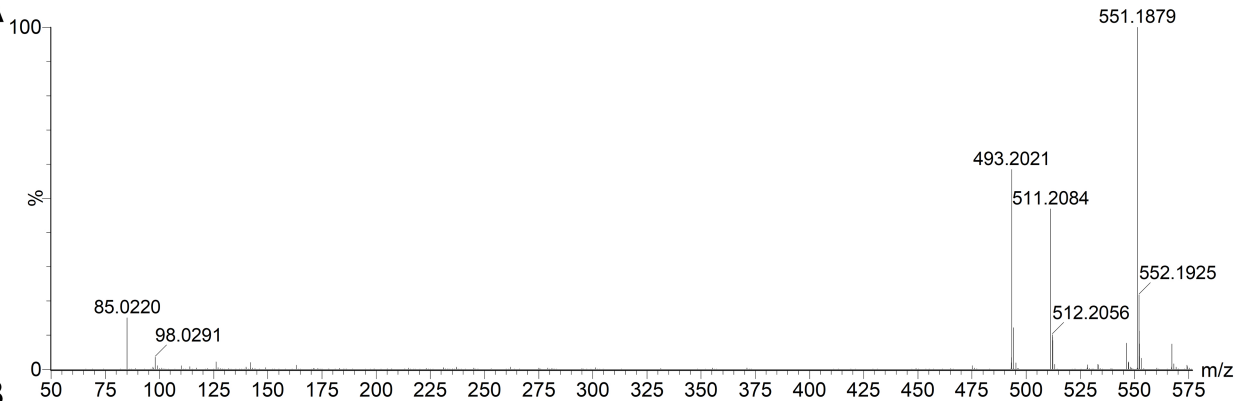**B**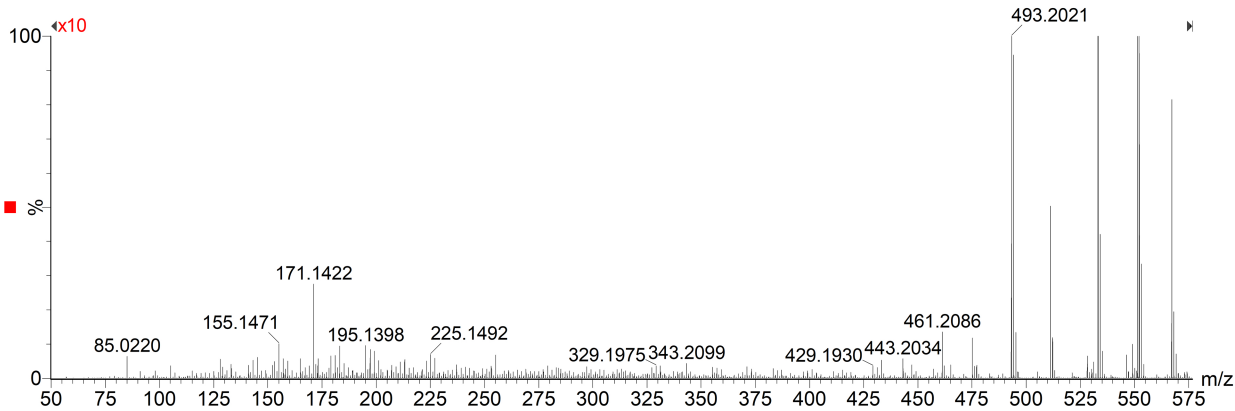

Supplement: SUPPLEMENTARY FIGURE 5 — MS spectra of physalin O and fragmentation pattern of the protonated molecular ion. (A) molecular ion peak of physalin O. (B) fragmentation pattern of protonated molecular ion. [file Image_5.PDF]

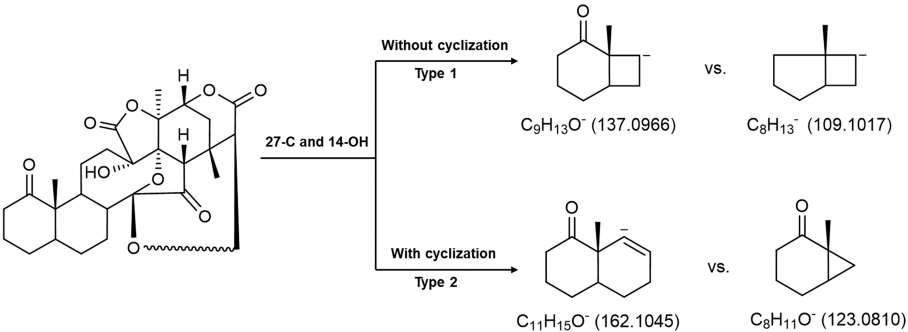

### Substituents in Frag-A

1. Alkenyl group: -2.0157
2. Oxhydril group: +15.9949
3. Epoxy group: + 13.9792
4. Methyl group: +14.0157
5. Cl: +33.9610

Supplement: SUPPLEMENTARY FIGURE 6 — Two fragmentation pathway in parent structure of physalins. [file Image_6.PDF]
