# Supplementary material for: Discovery of physalin biosynthesis and structure modification of physalins in Physalis alkekengi L. var. Franchetii
Source: Front Plant Sci. 2022 Oct 10;13:956083. doi: 10.3389/fpls.2022.956083 (PMC9589361; doi:10.3389/fpls.2022.956083)
Supplement: Supplementary file 9 [file Data_Sheet_3.docx]

# Supplementary Information

## MS behaviors of the physalin standards

The data acquisition mode of mass spectrometry was the data-independent acquisition (DIA) MS^E^. For the DIA MS^E^ mode, the primary mass spectrum of ions was obtained at the low CE channel, and fragment ion information was obtained at the CE channel. In theory, the fragment information of all ions can be obtained comprehensively without screening the parent ions in advance. The MS conditions were optimized using reference standards of physalins to achieve optimal MS sensitivity for detection and to obtain abundant fragment ions for structural elucidation. The negative ion mode provides more abundant structural information of physalins, while the positive ion spectra of physalins provide less information. Physalins showed higher response in [M+Na^+^] than [M+H^+^], which results in more interference with the analysis but also provides more abundant structural information.

### Fragmentation behaviors of physalin A

In positive ion mode, the parent ion of physalin A showed high response in 549.1710 (M+Na^+^), and fewer fragment ions were generated. In those fragment ions, fragmentation behaviors were split into neutral loss and cleavage of ring-open process (SF. 1).Fristly, the product ions with 531.1669, 509.1874, 491.1880, 473.1801 were the neutral loss of a seriesofH_2_O molecules, and the product ions with 549.1955, 431.1756 were the neutral loss of H_2_O, CO, and CO_2_ molecules. In addition, the product ions with 155.1471 were ring cleavage of Frag-A, which are a recognized signature fragment of physalin A. Notably, those fragment ions are also involved in [M+Na] ^+^and [M+H]^+^, such as 531.1669 (M+Na^+^-H_2_O) and 509.1874 (M+H^+^-H_2_O), which would make this analysis procedure difficult. The product ions with 171.1422 (C_9_H_15_O_3_^-^) are the Frag-B-(a) of physalin D, which is a recognized signature fragment of physalin.

### Fragmentation behaviors of physalin D

In positive ion mode, the parent ion of physalin D showed high response in 545.2097 (M+H^+^) and 567.1852 (M+Na^+^), and the response of 567.1852 was higher than 545.2097. In those fragment ions, fragmentation behaviors were split into neutral loss and cleavage of ring-open process (SF. 2).Firstly, the product ions with 527.2000, 509.1824, 491.1880 were the neutral loss of a seriesofH_2_O. The product ions with m/z 445.1904 were identified as molecules resulting from a neutral loss (-H_2_O) of C_9_H_9_O_2_^-^. The product ions with 473.1801 (C_25_H_29_O_9_^+^) were the neutral loss of C_2_H_4_ and CO_2_, which was ring cleavage of 26-carboxide and 24/25- C_2_H_4_ in Frag-B.Also, 445.1904, 427.1794 were series of the neutral loss (CO and H_2_O) of C_25_H_29_O_9_^+^.In addition, 155.1471 (C_11_H_20_O^+^) were ring cleavage of 9-C /11-C and 8-C/14-C in Frag-A, which is a recognized signature fragment of physalin A. The product ions with 171.1422 (C_9_H_15_O_3_^-^) are the Frag-B-(a) of physalin D, which is a recognized signature fragment of physalin.

### Fragmentation behaviors of the physalin G

In positive ion mode, the parent ion of physalin G showed high response in 527.2000 (M+H^+^) and 549.1710 (M+Na^+^), and the response of 549.1710 was higher than 527.2000. In those fragment ions, fragmentation behaviors were split into neutral loss and cleavage of ring-open process (SF. 3). The product ions with 509.1824, 491.1880 were the neutral loss of a seriesofH_2_O. and the product ions with 473.1801 and 419.0898 were the neutral loss of H_2_O, CO, and CO_2_ molecules. The product ions with 289.1575(C_16_H_17_O_5_^+^) were identified as molecules resulting from a neutral loss (-C_2_H_4_ and CO_2_) of Frag-B.The product ions with 171.1422 (C_9_H_15_O_3_^-^) were the Frag-B-(a) of physalin D, which is a recognized signature fragment of physalins.

### Fragmentation behaviors of the physalin L

In positive ion mode, the parent ion of physalin L showed high response in 551.1879 (M+Na^+^) and fewer fragment ions were generated. In those fragment ions, fragmentation behaviors were split into neutral loss and cleavage of ring-open process (SF. 4). Firstly, the product ions with 533.1796, 511.2084, 493.2021, 475.1950 were the neutral loss of a seriesofH_2_O molecules. In addition, the product ions with 173.1579, 155.1471 were ring cleavage of Frag-A, which is a recognized signature fragment of physalin L. Notably, those fragment ions are also involved in [M+Na] ^+^and [M+H]^+^, such as 533.1669 (M+Na^+^-CO) and 511.2084 (M+H^+^-H_2_O), which would make it difficult with this analysis procedure. The product ions with 171.1422 (C_9_H_15_O_3_^-^) is the Frag-B-(a) of physalin D, which is a recognized signature fragment of physalin.

### Fragmentation behaviors of the physalin O

In positive ion mode, the parent ion of physalin L showed high response in 551.1879 (M+Na^+^) and fewer fragment ions were generated. In those fragment ions, fragmentation behaviors were split into neutral loss and cleavage of ring-open process (SF. 5). Firstly, the product ions with 533.1796, 511.2084, 493.2021, 475.1950 were the neutral loss of a seriesofH_2_O molecules. Notably, those fragment ions are also involved in [M+Na] ^+^and [M+H]^+^, such as 533.1669 (M+Na^+^-CO) and 511.2084 (M+H^+^-H_2_O), which would make it difficult with this analysis procedure. The product ions with 171.1422 (C_9_H_15_O_3_^-^) are the Frag-B-(a) of physalin D, which is a recognized signature fragment of physalin.

## Summary: MS behaviors of the physalins

### The signature fragment of physalin.

Physalins are a group of natural ergosterol compounds with 13,14-ring-opening reaction and 16,24-cyclization structural characteristic. It is worth mentioning that the MS/MS product ions of physalin standards showed a signature fragment at 171.1422 (C_9_H_15_O_3_^-^) in positive ion mode, which is the structure of Frag-B (a) with 13,14-ring-opening. Base on this, the product ions with 171.1422 are recognized signature fragments of physalin.

### The signature MS behaviors of structure between 27-C and 14-OH

The parent ion spectra of physalin D and physalin G are [M+Na]^+^ and [M+H]^+^ at positive ion mode, and the parent ion spectra of physalin A, and physalin G are [M+Na]^+^ at positive ion mode.Based on this, the parent ion spectra in positive ion mode was used to distinguish whether annular structures are formed between 27-Cand 14-OH. Furthermore, physalin A, physalin D and physalin G showed a loss of C_2_H_4_andCO_2_, which is the signature neutral loss of annular structures between 27-CH_2_and 14-OH. And, two fragmentation pathways in parent structure of physalins, which corresponds to the product ion pairs with 149.0625 (C_9_H_9_O_2_^-^) vs. 121.0662 (C_8_H_9_O^-^), and 193.0852 (C_11_H_13_O_3_^-^) vs. 135.0456 (C_8_H_7_O_2_^-^) in negative ion mode. Of note, the product ion pairs with 149.0625 (C_9_H_9_O_2_^-^) vs. 121.0662 (C_8_H_9_O^-^) mainly showed in physalin A, physalin L and physalin O, and the product ion pairs with 193.0852 (C_11_H_13_O_3_^-^) vs. 135.0456 (C_8_H_7_O_2_^-^) mainly showed in physalin D and physalin G. This is due to the free 17-OH preferred form of C-8/C-14 alkenyl by loss of an H_2_O molecule in physalin, and ring-cleavage formed in C-9/C-10 and C6-C7 the same time.Then, A-ring opening processed via an RDA reaction which are summarized in SF. 6.

### The relationship between signature fragments and substituents of physalins

The differences among physalins mainly in the Frag-A were replaced with ring bearing substituents of differing electromagnetic properties. Generally speaking, losses of neutral fragments were associated with substituents of physalins. Firstly, the number of oxhydryls were promptly estimated by losses of H_2_O in the positive ion mode. Secondly, signature fragments were formed through binding between two major structural fragments at Frag-A of physalin and substituents, which are shown in fig.1.Besides, other substituents are also present in physalin, such as methoxyl, epoxy group and alkenyl. It is on this basis that signature fragments of Frag-A would be changed.
